# Development of Fully Human Antibodies Targeting SIRPα and PLA2G7 for Cancer Therapy

**DOI:** 10.3390/antib14010021

**Published:** 2025-03-03

**Authors:** Seungmin Shin, Du-San Baek, John W. Mellors, Dimiter S. Dimitrov, Wei Li

**Affiliations:** 1 Center for Antibody Therapeutics, Division of Infectious Diseases, Department of Medicine, University of Pittsburgh School of Medicine, Pittsburgh, PA 15261, USA; du-san.baek@glpg.com (D.-S.B.); jwm1@pitt.edu (J.W.M.); dimitrov666666@yahoo.com (D.S.D.); 2 GLPG US, 1401 Forbes Avenue, Pittsburgh, PA 15219, USA

**Keywords:** therapeutic antibody, human SIRPα, human PLA2G7, cancer, inflammatory disorders, cardiovascular diseases, immune modulation

## Abstract

**Background:** Macrophages play an important role in eliminating diseased and damaged cells through programmed cell death. Signal regulatory protein alpha (SIRPα) is a crucial immune checkpoint primarily expressed on myeloid cells and macrophages. It initiates a ‘do not eat me’ signal when engaged with CD47, which is typically expressed at elevated levels on multiple solid tumors. The phospholipase A2 Group 7 (PLA2G7), which is mainly secreted by macrophages, interacts with oxidized low-density lipoprotein (oxLDL) and associates with several vascular diseases and cancers. **Methods**: To identify potent fully human monoclonal antibodies (mAbs) against human SIRPα and PLA2G7, we conducted bio-panning of phage antibody libraries. **Results**: We isolated one human Fab (1B3) and VH (1A3) for SIRPα, as well as one human Fab (1H8) and one VH (1A9) for PLA2G7; the 1B3 Fab and 1A3 VH are competitively bound to SIRPα, interfering with CD47 binding. The 1B3 IgG and 1A3 VH-Fc augmented macrophage-mediated phagocytic activity when combined with the anti-EGFR antibody, cetuximab. The anti-PLA2G7 antibodies exhibited high specificity for the PLA2G7 antigen and effectively blocked the PLA2G7 enzymatic activity with half-maximal inhibitory concentrations (IC_50_) in the single-digit nanomolar range. Additionally, 1H8 IgG and its derivative bispecific antibody exhibited the ability to block PLA2G7-mediated tumor cell migration. **Conclusions**: Our anti-SIRPα mAbs are expected to serve as potent and fully human immune checkpoint inhibitors of SIRPα, enhancing the antitumor responses of SIRPα-positive immune cells. Moreover, our anti-PLA2G7 mAbs represent promising fully human PLA2G7 enzymatic blockade antibodies with the potential to enhance both anti-tumor and anti-aging responses. Anti-SIRPα and PLA2G7 mAbs can modulate macrophage phagocytic activity and inflammatory responses against tumors.

## 1. Introduction

The myeloid-specific immune checkpoint regulator SIRPα is a receptor for CD47, which is expressed on both tumors and normal tissues and represents a promising target for cancer immunotherapy [1,2,3]. The engagement of CD47 to SIRPα activates an antiphagocytic “do not eat me” signal in macrophages, dendritic cells, and neutrophils [4]. While CD47 has been explored as a cancer target [3,5], its ubiquitous expression throughout the human body has led to off-target effects, such as anemia [6,7]. Accordingly, the monoclonal antibody blockade against SIRPα could offer new treatment options for cancer by restoring macrophage-mediated phagocytosis without side effects associated with targeting CD47 directly [8,9,10].

PLA2G7, also known as platelet-activating factor acetylhydrolase (PAF-AH), is primarily secreted by macrophages and interacts with oxidized low-density lipoprotein (oxLDL) [11,12,13]. PLA2G7 hydrolyzes oxLDL into two bioactive products: lysophosphatidylcholine (lysoPC) and oxidized non-esterified fatty acids (oxNEFAs), leading to increased inflammation in human blood vessels. Elevated levels of PLA2G7 have been associated with various vascular diseases, and the therapeutic potential of PLA2G7 inhibition in these conditions has been documented [13,14]. Furthermore, recent studies have demonstrated that PLA2G7 is involved in immune-metabolic effects via regulating adipose tissue metabolism, and deletion of PLA2G7 in mice reduced age-related inflammation and extended lifespan [15,16]. Recent studies have also identified PLA2G7 as a pan-cancer diagnostic and prognostic marker [17,18]. PLA2G7 not only directly promotes tumor growth and migration [19], but also contributes to the immunosuppressive nature of the tumor microenvironment [20]. Notably, the immunosuppressive PLA2G7^high^ macrophages impeded CD8 T-cell function, and inhibition of PLA2G7 by darapladib enhanced the efficacy of anti-PD-1 antibody immunotherapy in hepatocellular carcinoma (HCC) mouse models [20]. These findings underscore the potential of blocking PLA2G7 enzymatic activity for cancer treatment and anti-aging interventions by inhibiting PLA2G7-mediated inflammation and reducing macrophage activation.

Here, we discovered both full-size and single-domain fully human antibodies targeting SIRPα. Our SIRPα mAbs competed with CD47 for binding to human SIRPα and exhibited a potent phagocytic effect when combined with the anti-EGFR antibody on colorectal cancer cell lines. We also identified VH and Fab antibodies against PLA2G7 and engineered a bispecific antibody by fusing the VH domain (1A9) to the C terminus of the IgG1 (1H8) heavy chain (HC). Our anti-PLA2G7 mAbs inhibited PLA2G7 enzymatic activity and reduced PLA2G7-mediated cell migration in colorectal cancer cell lines. Notably, the bispecific antibody demonstrated superior activity to 1H8 or 1A9 antibody monotherapy. Through these findings, we demonstrated the therapeutic potential of fully human anti-SIRPα and PLA2G7 mAbs for cancer therapy.

## 2. Materials and Methods

### 2.1. Preparation of Recombinant Human PLA2G7

Human PLA2G7 (residue 22–441) was synthesized in GenScript (Piscataway, NJ, USA) and then cloned into pCATDS-Fc plasmids via *NotI* and *AscI* restriction enzyme sites. The constructed plasmid DNA was amplified from *TOP10F’ E. coli* strain in the presence of 100 µg/mL of ampicillin and prepared using a Midi DNA-prep kit (Macherey-Nagel, 740410, Düren, Germany) for the transient transfection. Purified DNA was complexed with PEI-Max (Polysciences, 24765-1, Warrington, PA, USA) and applied to a culture of a Freestyle human embryonic kidney cell line (Gibco, A14527, Waltham, MA, USA). Six days post-transfection, PLA2G7-Fc was purified using affinity chromatography with protein A resin (Captiva, NC0997253, Santa Clara, CA, USA). The elution of bound proteins from protein A was performed by adding 100 mM of glycine buffer pH 3.0, and then the buffer was changed to phospho-buffered saline pH 7.4 (PBS) using a PD-10 desalting column (GE, 45-000-148, Marlborough, MA, USA). Protein purity was assessed using either SDS-PAGE or size exclusion chromatography (SEC). The concentration of protein was determined using a Nano Drop spectrophotometer 2000C (Thermo, ND2000C, Marlborough, MA, USA).

### 2.2. ICAT5 Fab and ICAT6 VH Libraries Panning Against SIRPα and PLA2G7

The biotinylated human SIRPα/CD172a-his (SIA-H82E0) and human CD47-his (CD7-H5227) were purchased from ACROBiosystems (Newark, DE, USA). The recombinant PLA2G7-Fc was biotinylated using EZ-Link Sulfo-NHS-SS-Biotin (Thermo, A39258, Waltham, MA, USA). The ICAT5 Fab and ICAT6 VH phage libraries constructed using human antibody gene repertoire from B cells were used in this study [21,22]. ICAT5 Fab and ICAT6 VH phage libraries were pre-blocked with 3% nonfat dry milk (Bio-Rad, 1706404, Hercules, CA, USA) in PBS (*w*/*v*) for 1 h at 25 °C. Blocked phages were incubated with 100 nM of biotinylated SIRPα-his (AcroBiosystems, SIA-H82E0, Newark, DE, USA) or 100 nM of biotinylated PLA2G7-Fc in the presence of a 10-fold excess of anti-HER2 IgG Pertuzumab (Selleckchem, A2008, Houston, TX, USA) to exclude Fc binder for 1 h at 25 °C. Bound phages were separated using streptavidin-coated magnetic beads (Invitrogen, 11-205-D, Carlsbad, CA, USA) and washed 10 times with 0.1% Tween-20 (*v*/*v*) in PBS at pH 7.4. Elution of bound phages was conducted by adding 1 µM of CD47 for SIPRα binders or 0.1 M of glycine at pH 2.5 for PLA2G7 binders. For the second and third rounds of panning, reduced concentration of biotinylated antigens (10 nM and 1 nM, respectively) and increased competitor ratio of anti-HER2 IgG were applied. After three rounds of panning, the binding of 192 individual clones was analyzed by ELISA, and selected clones were sequenced after plasmid rescue [21].

### 2.3. Purification of Fab, VH and IgG

The plasmid of positive clones was transformed into *HB2151 E. coli* competent cells, and then colonies were selected on ampicillin containing 2xTY plate for overnight incubation at 37 °C. The next day, a colony was inoculated into ampicillin containing 2xTY media and cultured in a shaking incubator at 30 °C. Isopropyl β-D-1-thiogalactopyranoside (IPTG) was added to the culture when OD_600_ reached between 0.4 and 0.6. The culture was relocated to a shaking incubator set at 30 °C and 200 rpm and incubated overnight. The next day, *E. coli* cells were harvested and resuspended in 1/10 volume of 0.5 mg/mL polymyxin B in PBS pH 7.4 and then incubated on the ice for an hour. The supernatant was collected by centrifugation at 12,000× *g* for 10 min and then loaded into pre-packed Ni-NTA resin. The bound Fab (his and flag-tagged) or VH (his and flag-tagged) were eluted by adding 300 mM of imidazole in PBS at pH 7.4, and then the buffer was changed to PBS at pH 7.4 using a PD-10 desalting column. For IgG or VH-Fc purification, the VH and VL of 1B3 or 1H8 cloned into pCATDS-HC and pCATDS-LC plasmid via *NotI/ApaI* and *NotI/BsiWI* restriction enzyme sites, and the 1A9 VH cloned into pCATDS-Fc plasmid via *NotI* and *AscI* restriction enzyme sites. The plasmid DNA was transfected into Expi293 cells and expressed for 6 days post-transfection. Expressed IgG or VH-Fc was purified by affinity chromatography with protein A resin. IgG or VH-Fc was eluted by adding 100 mM of glycine buffer at pH 3.0, and then the buffer was changed to PBS at pH 7.4 using a PD-10 desalting column.

### 2.4. Enzyme-Linked Immunosorbent Assay (ELISA)

SIPRα-his or PLA2G7-Fc proteins were coated on a 96-well plate (Corning, 3690, Corning, NY, USA) at 4 μg/mL in PBS overnight at 4 °C. The next day, plates were blocked with 3% nonfat dry milk in PBS for 2 h at room temperature (RT). Fab, VH, or IgG was incubated for 2 h at RT. Anti-FLAG mouse antibody (M2 clone)-HRP conjugated (Sigma, A8592, 1:2000 dilution, St. Louis, MO, USA) or anti-human kappa goat antibody-HRP conjugated (Invitrogen, A18853, 1:3000 dilution, Waltham, MA, USA) was added to detect binding of either Fab/VH or IgG. The 3,3′,5,5′-Tetramethylbenzidine (TMB) (Thermo, PI34028, Waltham, MA, USA) was added as a substrate, and then the enzymatic reaction was stopped by adding 2N sulfonic acid. Absorbance was read at 450 nm on an iMark microplate reader (Bio-Rad, Hercules, CA, USA).

### 2.5. Biolayer Interferometry (BLI)

The affinities of Fab, VH, IgG, or VH-Fc were analyzed using the biolayer interferometry BLItz (ForteBio, Menlo Park, CA, USA). Biotinylated-SIPRα-his or biotinylated-PLA2G7-Fc was mounted on the SA Biosensor (Satorius, 18-5019, Goettingen, Germany) for 2 min and equilibrated with PBS, pH  =  7.4 to establish baselines. Then, the anti-SIPRα or anti-PLA2G7 antibodies (125, 250, or 500 nM) were used for association for 2 min, and then the antibody was allowed to dissociate in PBS for 2 min. The background was established by running PBS instead of antibody in the association process, and the background curve was subtracted from the antibody sensorgram. The k_on_ and k_off_ were derived from sensorgram fittings and used for K_D_ calculation.

### 2.6. Cell Lines

Human histiocytic lymphoma U937, human colorectal carcinoma HCT116, and HT29 cells were purchased from ATCC. U937 and HT29 cells were maintained in RPMI (ATCC) supplemented with 10% (*v*/*v*) FBS (Gibco) and 1% (*v*/*v*) penicillin-streptomycin (P/S, Gibco, Waltham, MA, USA). HCT116 cells were maintained in DMEM (ATCC) supplemented with 10% (*v*/*v*) FBS and 1% (*v*/*v*) P/S. Primary NK cells were cultured in X-Vivo 10 medium (Lonza, 04-380Q, Basel, Switzerland), 5% (*v*/*v*) human AB serum (Corning), and human M-CSF (25 ng/mL, Biolegend, San Deigo, CA, USA).

### 2.7. Flow Cytometry Analysis

Human Seroblock (Bio-rad) was used to block the Fc receptor binding on the cell surface. The SIRPα expression of target cells was determined by staining an APC-conjugated anti-SIRP alpha antibody (Invitrogen, 17-1729-42). To confirm the cell surface binding of anti-SIRPα antibodies (1B3 IgG and 1A3 VH-Fc), cells were treated with antibodies (10 nM) for 1 h at 4 °C and then stained Alexa647-conjugated goat anti-human IgG (Invitrogen, A21445) for 0.5 h at 4 °C. To investigate the competitive binding between anti-SIRPα antibodies and CD47, cells were treated with antibodies (1, 10, or 100 nM) in the presence of biotinylated-human CD47 (500 nM) 1 h at 4 °C, and the residual CD47 binding was detected by using PE-conjugated streptavidin (Invitrogen, S866) (treatment with 0.5 h at 4 °C). Data were acquired using the flow cytometry BD FACScelesta (San Jose, CA, USA) and analyzed with FlowJo 10.7.1 (Tree Star, Minneapolis, MN, USA).

### 2.8. PLA2G7 Activity Assay

The blocking ability of anti-PLA2G7 mAbs against PLA2G7 enzymatic activity was determined using a PAF acetylhydrolase assay kit (Abcam, Ab133088, Cambridge, UK) [23,24]. The reaction buffer (0.1 M Tris, pH 7.2), 2-thio PAF, and dose-dependent anti-PLA2G7 mAbs were added to a 96-well plate. The mixture was incubated for 20 min at RT. The DTNB (5,5′-dithiobis-(2-nitrobenzoic acid) was added to develop the reaction and incubated for 1 min at RT. Absorbance was read at 415 nm on an iMark microplate reader (Bio-Rad).

### 2.9. Phagocytosis Assay

Monocytes were enriched from normal human peripheral blood mononuclear cells (PBMCs) (Zenbio Inc., Research Triangle Park, NC, USA) using a pan-monocyte cell isolation kit (Miltenyi Biotec, 130-096-537, Bergisch Gladbach, Germany). Monocyte-to-macrophage differentiation was performed by using X-Vivo 10 medium (Lonza, 04-380Q), 5% (*v*/*v*) human AB serum (Corning), and human M-CSF (25 ng/mL, Biolegend). Human colorectal cancer HCT116 or HT29 cells were labeled using a CellTrace CFSE Cell proliferation kit (Invitrogen, C34554) and added to the macrophage cultures in 96-well plates at 1 × 10^5^ cells per well. Anti-SIRPα 1B3 IgG (100 nM), 1A3-Fc (100 nM), or PBS control were added in the absence or presence of anti-EGFR cetuximab (10 nM) at varying concentrations and incubated at 37 °C for 3 h. Cells were harvested using Accutase (STEMCELL Technologies, Vancouver, BC, Canada), labeled with APC conjugated anti-CD14 mAb (Invitrogen, 17-0149-42), and then analyzed using flow cytometry. Phagocytosis was calculated as the percentage of CFSE-positive cells within the CD14-positive population.

### 2.10. Cell Migration Assay

The cells were plated in the upper chamber of a transwell plate (Corning, 3422) in 0% FBS media. The medium with 1% FBS was added to the bottom chamber. The recombinant PLA2G7 and anti-PLA2G7 mAbs were added to the upper chamber. PBS was used as vehicle control. The media was discarded and gently washed with PBS pH 7.4. The migrated cells on the lower side of the transwell membrane were stained/fixed by 0.5% crystal violet in 20% MeOH for 30 min at RT. The cells on the upper chamber of the membrane were scraped off using a cotton swab. The stained cells were washed with distilled water, dried, and then dissolved using a 10% acetic acid solution. Absorbance was read at 590 nm. The relative cell migration (%) was normalized based on the baseline (0%, blank plate control) and PLA2G7 activation (100%, PBS control + PLA2G7). The relative cell migration (%) = 100 × (A_590_^Sample^ − A_590_^blank^)/(A_590_^PBS control + PLA2G7^ − A_590_^blank^) [25,26].

### 2.11. Statistical Analysis

Data are represented as the mean ± s.d. of triplicate samples from one representative experiment based on at least three independent experiments unless otherwise specified. Comparisons of data from tests and controls were analyzed for statistical significance by a two-tailed, unpaired Student’s *t*-test using Microsoft Excel (Microsoft 365).

## 3. Results

### 3.1. Generation of Anti-SIRPα Antibodies with Competitive Epitopes to CD47

**Figure 1:**
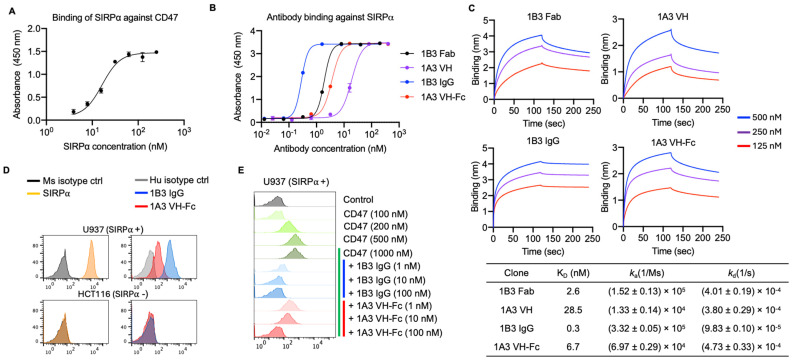
Generation and specificity of anti-SIRPα mAbs. (**A**) Binding of human SIRPα against human CD47. (**B**) Binding of anti-SIRPα mAbs (1B3 Fab, 1A3 VH, 1B3 IgG, and 1A3 VH-Fc) against recombinant human SIRPα. (**C**) Kinetics of anti-SIRPα mAbs (1B3 Fab, 1A3 VH, 1B3 IgG, and 1A3 VH-Fc) binding to human SIRPα, as measured by Blitz. (**D**) Left, the expression level SIRPα on human cancer cell lines, U937 (positive) and HCT116 (negative). Right, the cell surface binding of anti-SIRPα mAbs (10 nM of 1B3 IgG and 1A3 VH-Fc) on U937 and HCT116. (**E**) Inhibition of CD47 binding to U937 cells by anti-SIRPα mAbs (1B3 IgG and 1A3 VH-Fc). The residual bound CD47 level on the surface of the U937 cell was detected after competed by gradient concentration of anti-SIRPα mAbs. (**A**,**B**) Error bars represent the mean ± s.d. of triplicate samples from one representative experiment based on at least three independent experiments.

To identify fully human antibodies capable of blocking the human SIRPα/CD47 axis, we conducted the bio-panning of a phage-displayed antigen-binding fragment (Fab) library and a human immunoglobulin heavy chain variable domain (VH) library against the recombinant SIRPα. Before panning, we confirmed that SIRPα bound to recombinant CD47 with a two digits nanomolar affinity (half-maximal binding concentration EC_50_ = 20 nM), which is consistent with previous reports (Figure 1A) [27]. To select antibodies interacting with the CD47 binding epitope on SIRPα, we competitively eluted the anti-SIRPα binders with a micromolar (1 µM) range of recombinant CD47. We identified two candidates, one Fab (1B3) and one VH (1A3), which exhibited nanomolar range binding affinities (Figure 1B,C). The Fab 1B3 (EC_50_ = 1.9 nM and K_D_ = 2.6 nM) showed a higher binding affinity than the 1A3 VH (EC_50_ = 18.9 nM and K_D_ = 28.5 nM), as tested by ELISA and BLI. We then constructed bivalent versions: 1B3 IgG and 1A3 VH-Fc with L234A/L235A/P329G (LALA-PG) mutations on human IgG1 Fc region to eliminate the interaction with Fc receptors on macrophages. These bivalent constructs augmented binding strength due to avidity, showing approximately 10-fold higher affinity than their monovalent counterparts (Figure 1B,C). The 1B3 IgG (EC_50_ = 0.2 nM and K_D_ = 0.3 nM) exhibited a higher binding affinity than 1A3 VH-Fc (EC_50_ = 3.6 nM and K_D_ = 6.7 nM).

Both 1B3 and 1A3 demonstrated binding to the surface of SIRPα-positive U937 cell lines but did not bind to the SIRPα-negative HCT116 cell lines (Figure 1D), indicating their high binding specificity. We next assessed the capacity of 1B3 and 1A3 to block the binding of CD47 to its cognate receptor SIRPα. Both antibodies showed concentration-dependent inhibition of CD47 for binding to the U937 cell surface (Figure 1E). 1B3 IgG demonstrated superior potency in blocking CD47 binding compared to 1A3 VH-Fc. 1B3 IgG achieved complete blockade of CD47 binding at 10 nM, whereas 1A3 VH-Fc required a higher concentration of 100 nM to achieve the same effect (Figure 1E). Taken together, both 1B3 and 1A3 specifically bind to SIRPα and competitively inhibit CD47 binding, with 1B3 exhibiting a higher affinity and more potent blocking activity against CD47 binding compared to 1A3.

### 3.2. Identification of Two Anti-PLA2G7 Antibodies with Non-Overlapping Epitopes and Engineering a Bispecific Antibody

**Figure 2:**
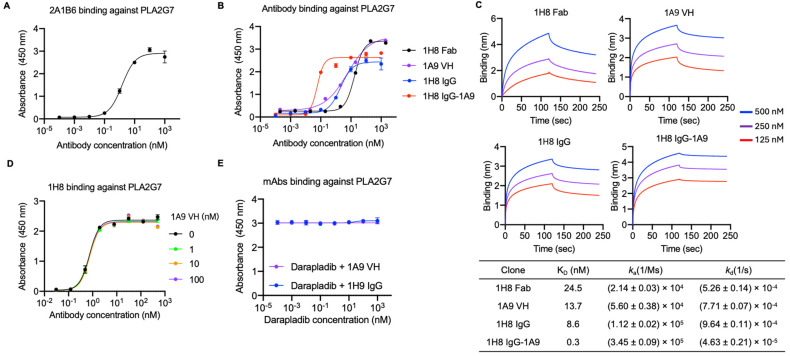
Generation and specificity of anti-PLA2G7 mAbs. (**A**) Binding of mouse anti-PLA2G7 mAb against in-house recombinant human PLA2G7-Fc. (**B**) Binding of anti-PLA2G7 mAbs (1H8 Fab, 1A9 VH, 1H8 IgG and 1H8 IgG-1A9) against human PLA2G7. (**C**) Kinetics of anti-PLA2G7 mAbs (1H8 Fab, 1A9 VH, 1H8 IgG, and 1H8 IgG-1A9) binding to human PLA2G7-Fc, as measured by Blitz. (**D**) Competitive binding of 1H8 IgG with 1A9 VH (0, 1, 10, or 100 nM) against human PLA2G7. The binding of 1H8 IgG antibody was detected. (**E**) Competitive binding of 1H8 IgG (100 nM) or 1A9 VH (100 nM) with PLA2G7 small molecule inhibitor, darapladib, against human PLA2G7. The binding of 1H8 IgG or 1A9 VH antibodies was detected. (**A**,**B**,**D**,**E**) Error bars represent the mean ± s.d. of triplicate samples from one representative experiment based on at least three independent experiments.

To identify anti-PLA2G7 antibodies, we constructed a fusion protein comprising PLA2G7 (Phe22-Asn441) linked to the N-terminus of human IgG1 Fc with a polypeptide linker (residues; GAP). We first validated the quality of this recombinant protein by demonstrating its binding to a commercial monoclonal human PLA2G7-specific mouse antibody, 2A1B6 (Figure 2A). After three rounds of panning using phage-displayed Fab library and VH library, we initially isolated 1H8 Fab (EC_50_ = 17 nM and K_D_ = 24.5 nM) and 1A9 VH clones (EC_50_ = 5.8 nM and K_D_ = 13.7 nM), both exhibiting high affinity to the recombinant PLA2G7 antigen as tested by ELISA and BLI (Figure 2B,C). We then reformatted Fab 1H8 into the IgG. The bivalent IgG 1H8 exhibited higher binding potency than Fab 1H8, as tested by ELISA (EC_50_ = 2.0 nM vs. 17 nM). Since the 1A9 VH did not compete with 1H8 for binding to PLA2G7 (Figure 2D), we engineered a bispecific antibody by fusing 1A9 VH to the C-terminus of 1H8 IgG HC with a (G_4_S)2 polypeptide linker, designated as 1H8 IgG-1A9. 1H8 IgG-1A9 showed enhanced affinity (EC_50_ = 0.05 nM) against PLA2G7 compared to the individual antibody (Figure 2B,C). Furthermore, we demonstrated that the epitopes of 1H8 IgG and 1A9 do not overlap with that of the well-established PLA2G7 inhibitor, darapladib, which binds to the catalytic triad of PLA2G7 [12] (Figure 2E). In summary, 1H8 and 1A9 specifically bind to PLA2G7 at distinct epitopes, both of which are separate from the catalytic triad.

### 3.3. Anti-SIRPα Antibodies Enhanced Macrophage Phagocytic Activity Against Cancer Cells When Combined with Tumor Opsonizing IgG1

**Figure 3:**
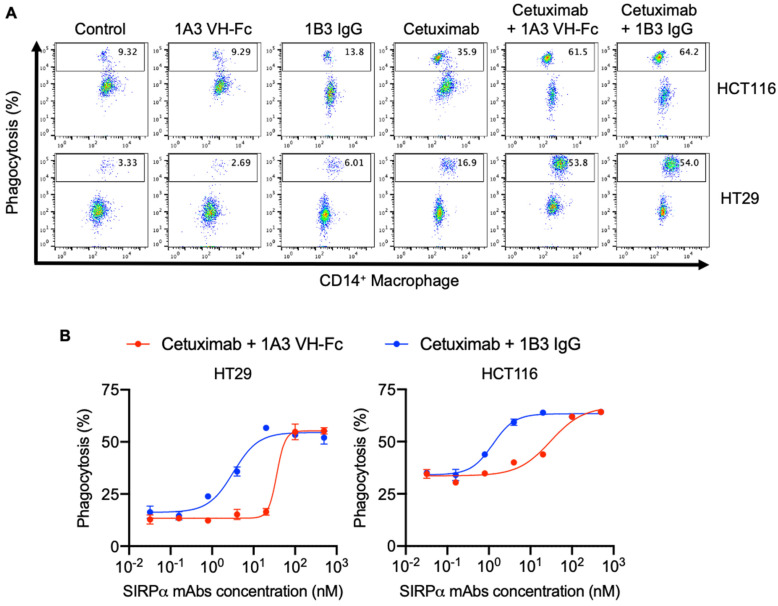
The restoration of phagocytosis function of anti-EGFR mAb by anti-SIRPα mAb. (**A**) Phagocytic activity of monotherapy or combined therapy of anti-SIRPα mAbs (100 nM) with anti-EGFR mAb (10 nM). The PBS was used as a control. The level of CFSE signal in CD14 positive macrophage was detected. (**B**) Dose-dependent phagocytic activity of anti-SIRPα mAbs with anti-EGFR mAb (10 nM). Error bars represent the mean ± s.d. of triplicate samples from one representative experiment based on at least three independent experiments.

The SIRPα/CD47 axis serves as a critical immune checkpoint, inhibiting macrophage-mediated phagocytosis of cancer cells. This “do not eat me” signal represents a significant barrier to effective anti-tumor immunity [5]. Since our anti-SIRPα antibodies are capable of blocking the SIRPα/CD47 interaction, we next sought to assess whether our antibodies can restore and enhance macrophage phagocytic activity against cancer cells. We designed a macrophage phagocytosis assay in which macrophages were differentiated from peripheral blood mononuclear cell-derived monocytes using macrophage colony-stimulating factor (M-CSF). These macrophages were then incubated with carboxyfluorescein succinimidyl ester (CFSE)-labeled tumor cells under different conditions. The extent of phagocytosis was quantified by measuring the percentage of CFSE+ tumor cells within CD14+ macrophages using flow cytometry. In this assay, we used the FDA-approved anti-EGFR mAb, cetuximab, in combination with EGFR-overexpressing colorectal cancer cell lines HCT116 and HT29. Cetuximab monotherapy exhibited discernable phagocytic activity due to the Fc/FcγR engagement (ADCP) [28], while anti-SIRPα mAbs alone showed no significant effect (Figure 3A). However, the combination of SIRPα mAb with cetuximab demonstrated significantly enhanced phagocytic activity compared to cetuximab monotherapy (Figure 3A). In the combination group, both 1B3 IgG and 1A3 VH-Fc exhibited concentration-dependent activity (Figure 3B). The EC_50_ of 1B3 IgG (3 nM on HT29 and 1 nM on HCT116) was significantly lower than that of 1A3 VH-Fc (36 nM on HT29 and 30 nM on HCT116), indicating the higher potency of 1B3 IgG (Figure 3B), which is consistent with the superior binding affinity and more effective competition against CD47 for 1B3 IgG compared to 1A3 VH-Fc. Taken together, we conclude that our anti-SIRPα mAbs effectively block the SIRPα-CD47 interaction, thereby enhancing the phagocytic activity induced by the anti-EGFR mAb on colorectal cancer cells.

### 3.4. Mono and Bispecific Antibodies Inhibited the PLA2G7 Enzymatic Activity and Blocked PLA2G7- Mediated Tumor Cell Migration

**Figure 4:**
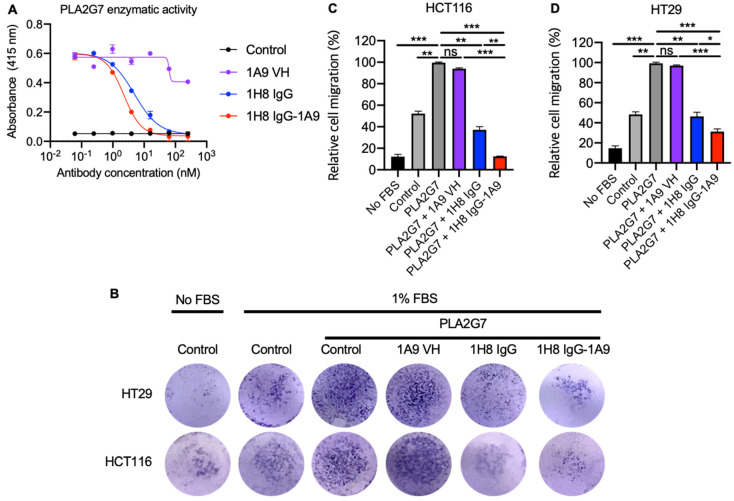
The blocking PLA2G7 enzymatic activity and cancer cell migration by anti-PLA2G7 mAbs. (**A**) Blocking of PLA2G7 enzymatic activity by anti-PLA2G7 mAbs. Error bars represent the mean ± s.d. (n = 3). (**B**–**D**) Blocking of cell migration by anti-PLA2G7 mAbs on colorectal cancer cell lines HT29 and HCT116. (**B**) Representative image showing the migrated cell on the lower side of the transwell membrane. (**C**,**D**) The relative cell migration (%) was normalized based on the baseline (0%, blank plate control) and PLA2G7 activation with PBS control (100%). Significance was determined by an unpaired two-tailed student’s *t*-test. * *p* < 0.05, ** *p* < 0.01, *** *p* < 0.001, ns means not significant. In (**B**), images are representative of three independent experiments. In (**C**,**D**), Error bars represent the mean ± s.d. of triplicate samples from one representative experiment based on at least three independent experiments.

PLA2G7 hydrolyzes oxidized phospholipids in LDL particles, and its enzymatic activity is associated with a risk of coronary heart disease and ischemic stroke [29]. Furthermore, PLA2G7 activity has also been linked to enhanced tumor cell migration and invasion [30]. PLA2G7 inhibition represents a promising therapeutic strategy for a plethora of indications, including cardiovascular diseases, aging, and cancers. We conducted a PAF acetylhydrolase assay to assess the blocking activity of anti-PLA2G7 mAbs against PLA2G7 enzymatic function. The 1H8 IgG effectively blocked PLA2G7 enzymatic activity despite not competing with darapladib for binding to PLA2G7 (Figure 2E and Figure 4A), while 1A9 VH-Fc did not show an inhibitory effect. Moreover, the bispecific antibody 1H8 IgG-1A9 (IC_50_ = 2.2 nM) exhibited slightly enhanced blocking activity compared to 1H8 IgG alone (IC_50_ = 4.8 nM) (Figure 4A). The higher blocking activity of 1H8 IgG-1A9 is consistent with its higher binding affinity to PLA2G7 (Figure 2B). Given that PLA2G7 has been reported to increase cancer cell migration, and PLA2G7 knockdown by siRNA led to reduced cancer cell migration [19], we next evaluated the inhibition of PLA2G7-mediated cell migration by our anti-PLA2G7 antibodies on both HCT116 and HT29 cells. Both 1H8 IgG and the bispecific antibody, 1H8 IgG-1A9, effectively inhibited cell migration, whereas 1A9 VH-Fc did not (Figure 4B–D). Moreover, the bispecific antibody1H8 IgG-1A9 exhibited higher inhibitory efficacy than 1H8 IgG1.

Taken together, our anti-PLA2G7 mAbs (1H8 IgG and 1H8 IgG-1A9) specifically bind to PLA2G7 and inhibit its enzymatic activity. Although 1A9 VH alone did not exhibit blocking activity, its incorporation into the bispecific antibody enhanced both the affinity and activity of 1H8 IgG.

## 4. Discussion

The SIRPα/CD47 axis represents a crucial innate immune checkpoint that inhibits the phagocytosis of tumor cells by macrophages and monocytes. This mechanism is commonly exploited by tumors to evade host innate immune surveillance. Blockade of this axis has emerged as a viable strategy to enhance anti-tumor immunity. Several anti-CD47 mAbs are currently undergoing clinical evaluation, including Hu5F9-G4 (magrolimab), ALX-148, TTI-621, and CC-90002 [31]. However, targeting CD47 directly can lead to undesirable side effects due to its widespread expression on normal cells, especially red blood cells. Consequently, developing antibodies that specifically block SIRPα has gained interest as a potentially safer and more targeted approach to unleash anti-tumor immune responses while minimizing off-target effects.

In this study, we generated and characterized two fully human mAbs targeting SIRPα: the Fab 1B3 and the single-domain antibody VH 1A3. Both mAbs showed high specificity and affinity for SIRPα, effectively competing with CD47 for receptor binding. This competitive inhibition serves as the underlying mechanism for their enhancement of macrophage phagocytosis [4]. Unlike other SIRPα-targeting mAbs [9,10], our antibodies are derived from fully human phage antibody libraries, potentially reducing the risk of immunogenicity when used in humans. Compared to the full-length Fab/IgG, the 1A3 single-domain VH antibody may offer different therapeutic profiles such as cost-effective manufacture, fast clearance PK, and enhanced penetration into solid tumor tissues [32,33] due to its smaller size. Furthermore, VH-Fc-based proteins may have different effector functions due to altered interaction with FcγR compared to the full-length IgG1. The comparison of the in vivo function of IgG1 and VH-Fc antibodies warrants further exploration.

Our anti-SIRPα antibodies alone did not augment macrophage phagocytic activity, a finding consistent with results from other groups demonstrating that anti-SIRPα antibody (BMS-986351) treatment alone exhibits limited efficacy [8], which may be attributed to the suboptimal interaction between SIRPα on macrophages and CD47 expressed on the colorectal tumor cells used in our study. Indeed, clinical studies have shown that CD47/SIRPα blockade antibodies as monotherapy lead to limited therapeutic responses [34]. The engagement of CD47 activates SIRPα intracellular tyrosine inhibition motifs, recruiting tyrosine phosphatases 1 and 2 (SHP1 and SHP2), which can dephosphorylate FcγR ITAM on macrophages [8]. Therefore, to maximize the enhancement of macrophage phagocytosis, CD47/SIRPα blockade antibodies can be combined with tumor-opsonizing therapeutic antibodies that mount pro-phagocytic signaling through Fc/Fcγ interactions. Combination therapy using magrolimab with rituximab or cetuximab has exhibited benefits in treating relapsed/refractory non-Hodgkin lymphoma (R/R NHL) or advanced colorectal cancer, respectively, in phase Ib/II studies, demonstrating promising overall response rates (ORRs) and partial response (PR) rates [7,35]. In our in vitro study, we demonstrated that co-treatment with our anti-SIRPα antibodies and cetuximab significantly enhanced macrophage phagocytic activity in a concentration-dependent manner (Figure 3). Further investigation of their synergistic effects in vivo is warranted. Developing bispecific antibodies that combine anti-SIRPα mAbs with tumor opsonizing mAbs, such as anti-EGFR, represents a promising approach [36,37,38]. These bispecific antibodies could block the SIRPα-CD47 interaction and directly target cancer cells, potentially enhancing anti-cancer effects and mitigating resistance mechanisms. Furthermore, our anti-SIRPα antibodies could potentially be combined with other cytotoxicity-stimulating agents, including T cell immune checkpoint inhibitors such as anti-PD-1 and anti-CTLA-4 antibodies [39]. Additionally, they might be used in conjunction with Azacitidine, which can upregulate the expression of calreticulin, an ‘eat me’ signal, on tumor cell surfaces [40].

Recently, PLA2G7 has emerged as a promising target for therapeutic intervention due to its multifaceted roles in various pathological processes, particularly in cardiovascular diseases, aging, and cancers. The PLA2G7 enzymatic activity generates bioactive lipid mediators, which play roles in atherosclerosis and tumor progression [11,12,13,14]. Elevated PLA2G7 activity has been associated with increased tumor cell migration, invasion, and metastasis in various cancer types [19]. PLA2G7 inhibition by siRNA could alter the immunosuppressive tumor microenvironment, potentially enhancing the efficacy of T cell immune checkpoint blockade antibodies [19]. While small molecule inhibitors of PLA2G7 (like darapladib) have shown limited success in clinical trials [41], antibody-based approaches may offer improved efficacy and safety profiles due to the antibody′s high potency and specificity. In this study, we developed and characterized two fully human mAbs targeting PLA2G7. The mAbs, 1H8 IgG, and 1A9 VH-Fc demonstrated high specificity binding to distinct epitopes on PLA2G7, with 1H8 IgG showing significant inhibition of PLA2G7’s enzymatic function. The bispecific antibody 1H8 IgG-1A9 exhibited even greater inhibitory activity, indicating that combining different mAb fragments with distinct epitopes can enhance the therapeutic potential. Our antibodies did not compete with darapladib for binding to PLA2G7 (Figure 2E), suggesting that their epitopes are located outside the enzyme’s catalytic site. This observation indicates a unique mechanism by which these antibodies inhibit PLA2G7’s enzymatic activity, which needs to be further explored by structural and epitope mapping studies.

One of the main challenges in targeting PLA2G7 with small-molecule inhibitors like darapladib is their short half-life (~30 h) [42], which necessitates frequent dosing and may limit their long-term efficacy in cardiovascular disease and cancer therapy [41]. In contrast, mAbs generally have longer half-lives, potentially providing more sustained target inhibition. However, the application of PLA2G7 inhibition in cancer therapy is still in its early stages, and there are concerns about the ability of inhibitors to effectively target and modulate cancer-associated PLA2G7 activity in vivo [19,43,44]. Given PLA2G7’s role in promoting cancer cell migration [17], the anti-migratory effects observed with 1H8 IgG and 1H8 IgG-1A9 in our study are particularly encouraging. These findings suggest that PLA2G7-targeting mAbs could potentially be developed as novel anti-cancer agents. However, further studies are needed to fully elucidate the mechanisms by which PLA2G7 contributes to cancer progression and to optimize the therapeutic efficacy of these mAbs.

## Data Availability

Human SIRPα and PLA2G7 antibodies are available under a material transfer agreement or research collaborative agreement.
